# Long-Range Underwater Communication Based on Time Reversal Processing Using Various Diversity Methods

**DOI:** 10.3390/s22093138

**Published:** 2022-04-20

**Authors:** Donghyeon Kim, Jeasoo Kim, Jooyoung Hahn

**Affiliations:** 1 Department of Convergence Study on the Ocean Science and Technology, Korea Maritime and Ocean University, Busan 49112, Korea; donghyeon.ual@gmail.com; 2 Department of Ocean Engineering, Korea Maritime and Ocean University, Busan 49112, Korea; 3 Agency of Defense Development, Changwon-si 51682, Korea; hahnjy@add.re.kr

**Keywords:** time reversal, diversity, deep water, communication

## Abstract

Time reversal processing (TRP) exploits signal diversity methods, namely, spatial, temporal, beam, and frequency, to mitigate the distortion caused by multipath time delay. Using the same experimental data, this study compares the performance of communication utilizing TRP based on various diversity methods. In October 2018, the biomimetic long-range acoustic communication experiment 2018 (BLAC18) was conducted in the East Sea, east of Pohang, Korea. During the experiment, communication signals modulated by binary phase-shift keying were transmitted over a range of 60 km, and a vertical line array of 16 elements (with an aperture of ∼42 m) was utilized. The BLAC18 analysis showed that the performance of each diversity method depends on the order of diversity. When the order of diversity was one, the beam diversity method with the beamformed signal yielded the best performance. For the maximum order of diversity, however, the spatial diversity method delivered the best performance, owing to the high channel variability and large number of receivers.

## 1. Introduction

Long-range communication is a challenging task requiring systematic research because of its unstable performance, which is caused by an increase in delay spread, a reduction in channel capacity due to rising transmission loss with range, and channel fluctuation. Recently, in conjunction with the development of autonomous underwater vehicles that cruise over a long range (of the order of hundreds of kilometers), long-range communication technology has attracted growing interest, with numerous experimental studies being conducted to develop a stable long-range underwater communication system.

Stojanovic demonstrated the feasibility of long-range communication for the first time using experimental data from the Woods Hole Oceanographic Institution’s long-range (200 km) underwater communication experiment [1,2]. During the experiment, communication signals designed with higher order constellations such as 8-quadrature amplitude modulation (QAM) and 8-phase shift keying (PSK) were transmitted. In 1998, Plaisant demonstrated communication signals designed by spread spectrum techniques as well as PSK modulation with two experimental data sets (50 km: PSK modulation, 20 km: spread spectrum) [3]. To mitigate the intersymbol interference (ISI) caused by multipath time delay, the aforementioned results were analyzed based on a multichannel decision feedback equalizer (M-DFE). This approach is necessarily highly complex computationally, and this complexity is proportional to the length of the equalizer in taps and the number of array elements [4].

To effectively address this problem, time reversal processing (TRP), which enables self-equalization and utilizes diversity to increase the channel capacity, has been utilized as an alternative to M-DFE for long-range communication [4]. TRP is calculated by correlating the received signal with the channel impulse response, which improves the signal-to-noise ratio (SNR) and mitigates the ISI by utilizing multipath time delay that can degrade communication performance. Parvulescu and Clay [5] visually described the improvement of the SNR in a one-transmitter one-receiver environment. The performance of the TRP can be improved using a diversity method [6]. Four types of diversity methods exist, namely, spatial, temporal, beam, and frequency, and they operate on the principle of coherently combining multiple TRP outputs. TRP, which is the precombining process, is a special case of reduced-complexity M-DFE [4].

Since 2008, the Japan Agency for Marine-Earth Science and Technology (JAMSTEC), the institution that has conducted the most long-range underwater acoustic communication experiments to date, has been conducting experiments over various ranges (30–1450 km) and using various modulation methods while investigating multiuser communication [7,8,9,10,11]. JAMSTEC has transmitted communication signals designed up to 16-QAM, which is the highest-order modulation method designed for long-range underwater communication. They analyzed these signals using spatial diversity-based TRP with a vertical line array (VLA). Song presented and compared the results of a basin-scale (∼3250 km) tomography experiment conducted in November 1994 based on spatial and temporal diversity methods [12,13]. Furthermore, unlike other long-range experiments using a VLA, a long-range acoustic communication (LRAC10) experiment was conducted in 2010 at a range of up to 550 km using a horizontal line array [14,15,16,17,18]. A beam diversity-based TRP with an azimuth angle was utilized to evaluate the performance of communication signals that were designed using various modulation methods such as PSK modulation [14,15], OFDM [16], and spread spectrum [18].

The purpose of this study is twofold. First, this paper describes the experiment conducted in the East Sea. The East Sea area is located east of Pohang, Korea, and is a so-called miniature ocean (mini-ocean) because it has an independent deep convection system, which is a characteristic of oceans, and its circulation system and hydrography are similar to that of major ocean basins. The dynamics and oceanographic characteristics of this area can be predicted in advance [19,20,21]. Thus, experiments in the East Sea have oceanographic value. Furthermore, conducting long-range experiments is difficult because they are resource- and time-consuming endeavors, and are performed only by a small number of institutions [1,7,12,14,22]. Therefore, the East Sea long-range communication experiment itself, which is the first experiment conducted using various long-range communication signals in Korea, is very meaningful. The experiment was named the biomimetic long-range acoustic communication experiment 2018 (BLAC18). Second, this paper compares the performance of various diversity methods. During the BLAC18 experiment, communication signals were transmitted several times and obtained using a VLA. Using this experimental data, Park confirmed that combining two diversity methods rather than using a single diversity method improved the communication performance [23]. Kim presented the results of the beam diversity method with a vertical line array, notably the results of a performance comparison based on various beam combinations [24]. The performance of the diversity method depends on the channel variability, and we analyze and compare the communication performance according to the type and order of the diversity method in terms of this variability. However, a study involving a comparison of the results of various diversity methods for the same data based on the order of diversity has not been reported thus far. The contribution of this study is therefore to provide a comprehensive comparison of the communication performance and the dependence thereof on the order of diversity and the type of diversity.

The remainder of this paper is organized as follows. Section 2 reviews the TRP formulation using various diversity methods. Section 3 outlines the experiment conducted in the East Sea off the coast of Pohang, Korea, and describes the structure of the communication signal used as the basis for analysis in this study. Section 4 presents and compares the results of our analysis of the long-range communication performance for various diversity methods with the theories introduced in Section 2. Finally, concluding remarks are stated in Section 5.

## 2. Review of TRP with Various Diversity Methods

As mentioned above, TRP is the correlation between a received signal and the channel impulse response and can be extended using a diversity method. The diversity method improves the SNR by coherently combining multiple TRP outputs. For example, the spatial diversity method combines the TRP outputs acquired from different locations. The reader can apply the appropriate diversity method according to the experimental conditions, such as the type of signal, the number of transmissions, and the number of receivers. In this section, we review the theoretical TRP formulation using three diversity methods (spatial, temporal, and beam). As we only transmitted modulated signals with a single carrier frequency, an analysis was not conducted using the frequency diversity method. Equation (1) represents the TRP output using both the spatial and temporal diversity methods [6], where the inside structure of ∑ denotes the TRP output when the order of diversity is one.

(1) $$Y\left(\omega \right)=\sum_{m\;or\;n=1}^{M\;or\;N}R_{m\;or\;n}\left(\omega \right)H_{m\;or\;n}^{*}\left(\omega \right)=S\left(\omega \right)\left[\sum_{m\;or\;n=1}^{M\;or\;N}H_{m\;or\;n}\left(\omega \right)H_{m\;or\;n}^{*}\left(\omega \right)\right],$$

where S(ω) and Y(ω) are the source signal and the TRP output using the spatial or temporal diversity method, respectively. R(ω) and H(ω) represent the received signal and the channel impulse response, respectively, and an asterisk (or ()*) denotes a complex conjugate. The order of diversity varies depending on the diversity method. In Equation (1), *M* and *N* denote the order of diversity of the spatial and temporal methods, respectively. Because the spatial diversity method-based TRP utilizes the received signals measured from several receivers, the order of spatial diversity (*M*) is the same as the number of receivers. In the temporal diversity method, which utilizes multiple transmissions measured from a single receiver, the order of temporal diversity (*N*) is the same as the number of transmissions.

**Figure 1:**
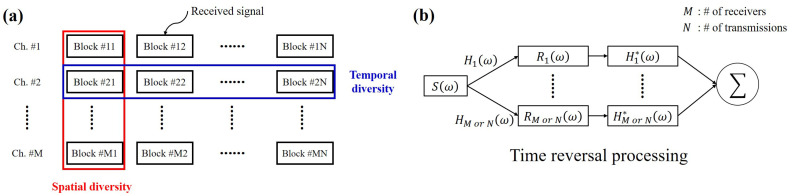
(**a**) Schematic of the methods used to select the received signals. The spatial and temporal diversity methods utilize the received signals in the red and blue boxes, respectively. (**b**) Block diagram for passive time reversal processing (TRP) using the spatial or temporal diversity method.

Figure 1a shows a schematic of the method used to select the received signals for the spatial and temporal diversity methods. The black boxes indicate multiple transmissions obtained from the array, i.e., all the data acquired during the experiment. The spatial and temporal diversity methods utilize the received signals in the red and blue boxes, respectively. The overall system based on these diversity methods is shown in Figure 1b.

**Figure 2:**
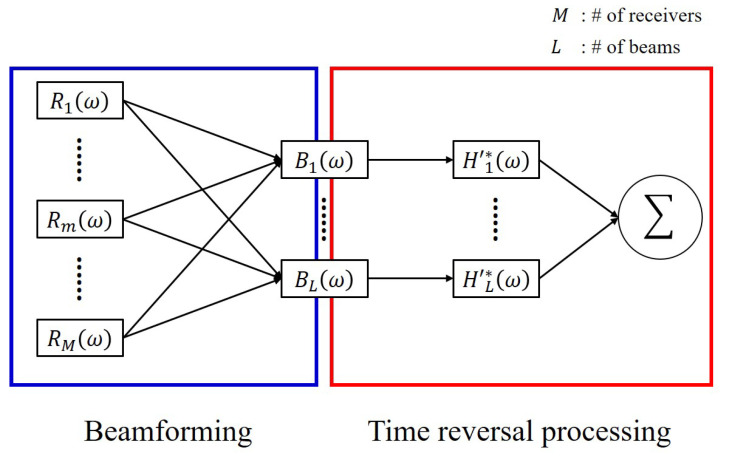
Block diagram illustrating time reversal processing using the beam diversity method. The beam diversity method has two processes: (1) beamforming the received signals (blue box) and (2) estimating the channel impulse response of the beamformed signal and using this response for time reversal processing (red box).

Whereas the spatial and temporal diversity methods utilize the received signals, the beam diversity method utilizes the signals obtained by beamforming the received signals. The overall system based on the beam diversity method is shown in Figure 2, and its mathematical formulations are shown in Equations (2)–(6):

(2) $$B_{l}\left(\omega \right)=R\left(\omega \right)W_{l}^{H}\left(\omega \right)=S\left(\omega \right)H\left(\omega \right)W_{l}^{H}\left(\omega \right)=S\left(\omega \right)H_{l}^{\prime }\left(\omega \right),$$

(3) $$\begin{array}{c} R\left(\omega \right)=\left[R_{1}\left(\omega \right)\ldots R_{m}\left(\omega \right)\ldots R_{M}\left(\omega \right)\right],\;H\left(\omega \right)=\left[H_{1}\left(\omega \right)\ldots H_{m}\left(\omega \right)\ldots H_{M}\left(\omega \right)\right],\\ W_{l}\left(\omega \right)=\left[W_{l}^{1}\left(\omega \right)\ldots W_{l}^{m}\left(\omega \right)\ldots W_{l}^{M}\left(\omega \right)\right], \end{array}$$

(4) $$W_{l}^{m}\left(\omega \right)=\exp\left[-i\omega \left(m-1\right)\frac{d}{c}\sin\theta_{l}\right],$$

(5) $$Y^{\prime }\left(\omega \right)=\sum_{l=1}^{L}B_{l}\left(\omega \right)H_{l}^{\prime *}\left(\omega \right)=S\left(\omega \right)\left[\sum_{l=1}^{L}H_{l}^{\prime }\left(\omega \right)H_{l}^{\prime *}\left(\omega \right)\right],$$

where *d*, *c*, and $\theta_{l}$ denote the spacing between adjacent receivers, the speed of sound, and the angle of the lth path, respectively; ${\left(\right)}^{H}$ denotes the Hermitian transpose.

In Equation (2), $B_{l}\left(\omega \right)$ is calculated by steering the received signals to the angle of the lth path and is defined as a beamformed signal [25,26]. H(ω) and R(ω) in Equation (3) represent the channel impulse responses between the source and the array and the received signals obtained from the array, respectively. In this study, as a vertical line array is utilized, R(ω) and H(ω) have *M* components. $W_{l}\left(\omega \right)$ in Equation (3) represents the steering vector in the direction of the angle of the lth path. $B_{l}\left(\omega \right)$ can be expressed as the product of S(ω) and $H_{l}^{\prime }\left(\omega \right)$ if R(ω) is separated into S(ω) and H(ω) (Equation (2)). $H_{l}^{\prime }\left(\omega \right)$, the product of H(ω) and $W_{l}\left(\omega \right)$, represents the channel impulse response of the beamformed signal along the lth path. In Equation (5), $Y^{\prime }\left(\omega \right)$ represents the TRP output using the beam diversity method and has a structure similar to Equation (1). Equations (2) and (5) correspond to the part marked “Beamforming” (blue box) and the part marked “Time reversal processing” (red box) in Figure 2, respectively. A comparison of Figure 1b and Figure 2 reveals two main differences: (1) The first step of the beam diversity method is to beamform (or steer) the received signals (blue box in Figure 2); (2) In the TRP step (red box in Figure 2), these beamformed signals are used instead of the received signals. Therefore, in the beam diversity method, the order of beam diversity is the same as the number of beams.

## 3. BLAC18 Experiment

**Figure 3:**
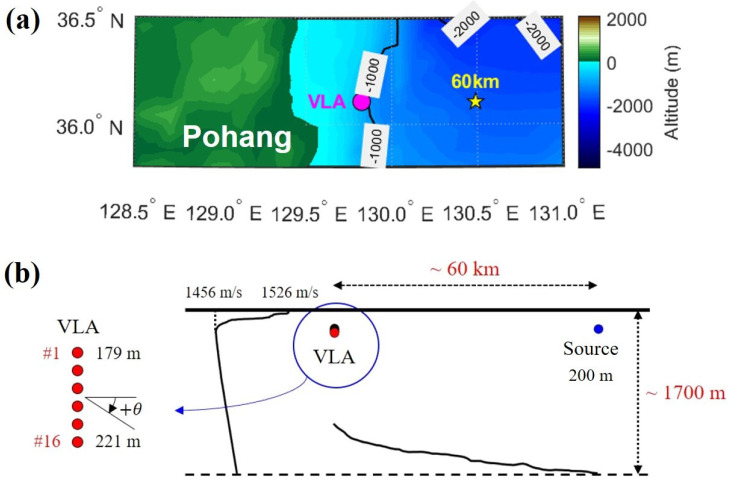
(**a**) Bathymetry of the experimental area in the East Sea. Depth contours are in meters. The biomimetic long-range acoustic communication 2018 (BLAC18) experiment was conducted in October 2018. The five-point star indicates the source location and the magenta circle indicates the VLA location. (**b**) Schematic of the BLAC18 experimental setup. The red and blue solid circles indicate the receivers and sources, respectively. A 16-element, 42-m-long VLA moored to the sea floor in water with an approximate depth of 950 m recorded the communication signals. A positive angle denotes an upward ray path. The sound-speed profile was determined by measuring the CTD.

In October 2018, BLAC18 was conducted in the East Sea, east of Pohang, Korea. At the site of the experiment, the water depth was in the range of 950–1500 m. The communication signals were transmitted over a range of 60 km. The VLA consisted of 16 elements spanning a 42-m aperture with an element spacing of 2.8 m. In the experiment, the source depth was 200 m and the VLA covered a depth range of 179–221 m at a water depth of approximately 950 m. Figure 3a shows the experimental area. The five-pointed star indicates the source location and the magenta circle indicates the VLA location. The depth contours represent the depth in meters.

A schematic of the experiment is shown in Figure 3b. The sound-speed profile displayed in Figure 3b was obtained by measuring the conductivity, temperature, and depth (CTD) at the VLA location, which features an underwater sound channel with an acoustic axis at a depth of 250 m. In Figure 3b, the red and blue solid circles indicate the receivers and sources, respectively. A positive angle θ is defined for an upward path. This experiment was conducted in collaboration with the Korea Institute of Ocean Science & Technology (KIOST). The KIOST-operated R/V Ieodo was used for equipment deployment/recovery. Iridium and depth sensors were used to track the location and depth of the VLA, respectively.

**Figure 4:**
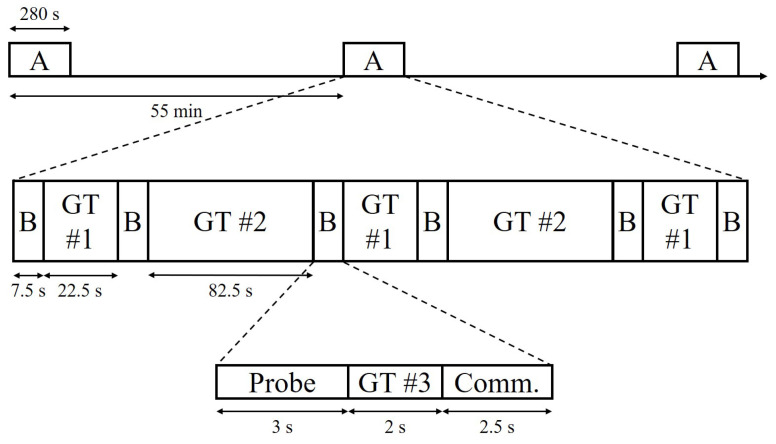
Communication signal transmitted by the source during BLAC18. Each block denoted by the letter {A} (280-s long), consisting of six data packets denoted by the letter {B} (7.5-s long), was repeated every 55 min. Each data packet {B} consists of an LFM signal as a channel probe and a communication sequence. Blocks labeled {GT#1∼3} represent guard times that are 22.5-, 82.5-, and 2-s long, in this order.

This paper presents an analysis of the data transmitted during a 2-h period. The detailed structure of the transmitted signal is illustrated in Figure 4. Each block denoted by the letter {A} (280 s long) consists of six data packets denoted by the letter {B} and two types of guard times, which are indicated as {GT#1} and {GT#2} and have lengths of 22.5 and 82.5 s, respectively. Each data packet {B} is 7.5 s long and consists of a linear frequency modulated (LFM) signal as a channel probe and a communication sequence. The 280-s long signal was repeated every 55 min. A total of 18 data packets were transmitted during the 2-h period. During the BLAC18 experiment, many click sounds were recorded. Three data packets were highly contaminated owing to the click sounds and were excluded from the analysis.

The probe signal was an LFM chirp with a Hanning window having a duration and frequency of 3 s and 2.2–2.9 kHz, respectively. The communication signal consisted of a total of 1255 symbols (2.47 s transmission duration), modulated by BPSK with a bit rate of 512 bits/s. The shaping pulse was a square-root raised cosine filter with a roll-off factor of beta = 0.25 and the carrier frequency was 2560 Hz. Among the 1255 symbols, the first 255 are m-sequence signals designed for Doppler estimation and synchronization. A 2-s-long guard time, indicated as {GT#3} in Figure 4, is included between the probe and communication signals.

## 4. Experimental Results

### 4.1. Order of Beam Diversity Method

**Figure 5:**
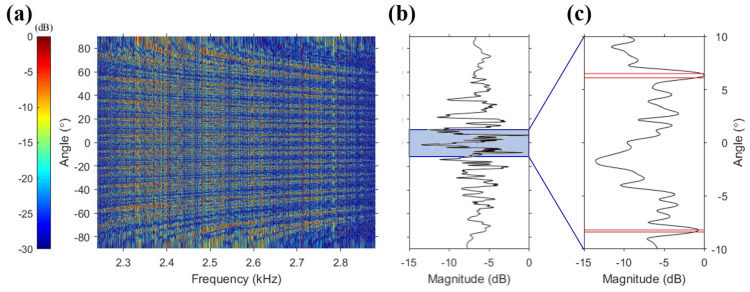
Conventional beamforming output using one data packet; (**a**) beamformed output as functions of the frequency (Hz) and grazing angle (deg). Aliasing occurred because of the carrier frequency, which is approximately 10 times the design frequency. (**b**) Beamformed output incoherently summed over the frequency band. (**c**) Enlargement of (**b**) from $-5^{\circ }$ to $15^{\circ }$. The red lines represent the angles of all paths estimated from the channel impulse response (Figure 6). The angles cannot be estimated from beamforming because of aliasing and beam resolution.

**Figure 6:**
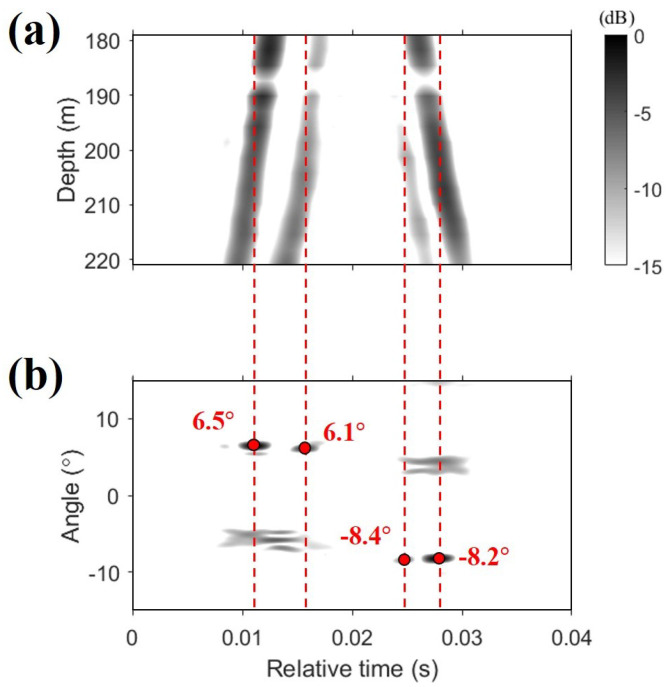
(**a**) Channel impulse response estimated at a range of 60 km from a linear frequency modulated (LFM) channel probe after matched-filtering. Four dominant paths are identified. (**b**) Beam-time migration of the channel impulse response corresponding to (**a**), respectively. The red dashed lines between the channel impulse response and the beam-time migration represent the relative time delays of the dominant paths, and the red circles on the beam-time migration plot represent the relative time delays and angles of the dominant paths.

The orders of the spatial and temporal diversity methods were 16 (the number of receivers) and 15 (=18 − 3, the number of packets), respectively. In the beam diversity method, the order of diversity is the number of dominant paths. In general, this is estimated by beamforming the received signals. However, because the carrier frequency was approximately 10 times the design frequency, the number of dominant paths was not estimated owing to aliasing. Additionally, the beam resolution, i.e., $0.89\frac{\lambda }{D}$ (rad) in a line array [27], determined by the wavelength and array aperture is $0.7^{\circ }$ under our experimental conditions ($f_{c}=2560\;\mathrm{Hz},D=42\;m$), and adjacent paths lower than the beam resolution cannot be separated. Figure 5a shows the conventional beamforming output according to the frequency, with aliasing occurring as expected. Figure 5b shows the conventional beamforming output averaged over the frequency band. An enlargement of the region from $-5^{\circ }$ to $15^{\circ }$ of Figure 5b is shown in Figure 5c, in which the red lines correspond to the angles of the path obtained from Figure 6. As is evident from the figure, the angles of all dominant paths cannot be estimated by the sidelobe because of aliasing and beam resolution. Therefore, to obtain the angles of all dominant paths, we used the channel impulse response estimated using the probe signal.

The channel impulse response is shown in Figure 6a, from which it is clear that four dominant paths exist. Estimation of the channel impulse response ensures the separation of multiple arrivals in the beam-time domain, referred to as “beam-time migration,” through conventional beamforming. Figure 6b shows the beam-time migration of the channel impulse response. The red dashed lines between Figure 6a,b represent the relative time delays of dominant paths and are shown to extract the angles from the beam-time migration. The process whereby the angles are extracted is as follows: (1) Identify locations with values above a certain threshold in the beam-time domain. At this time, the value of the threshold varies with the selected data. (2) When two or more locations have the same time, one location is selected considering the path direction, that is, the sign of the angle, or the level corresponding to the path from the channel impulse response. The relative arrival time and angle information of the dominant paths are indicated with the red circles in Figure 6b, and these angles were used for the beam diversity method. Figure 6 shows the channel impulse response and beam-time migration estimated from one data packet, and the number of dominant paths was maintained in the same range.

### 4.2. Impact of Diversity

**Figure 7:**
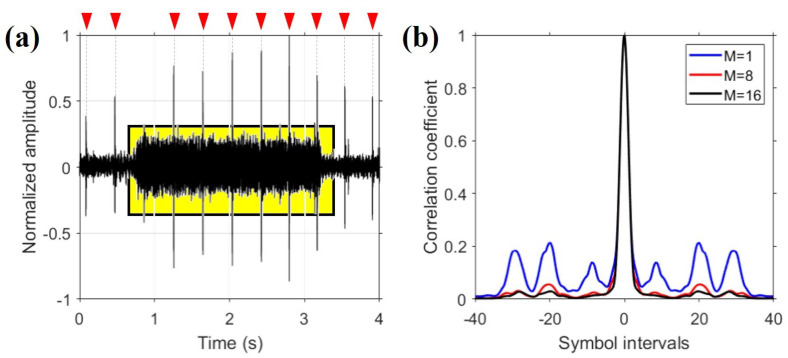
(**a**) Signal obtained by one receiver. The click sounds captured during the experiment are identified by red arrows. (**b**) Averaged Q-function as a function of the order of spatial diversity. The blue, red, and black lines correspond to orders of spatial diversity (*M*) of 1, 8, and 16, respectively. As the order of diversity increases, the sidelobes become smaller, i.e., the Q-function converges to the delta function.

The TRP performance is improved with a diversity method [6]. In this section, we reproduce the work conducted in a previous study [6] to explain the effect of the diversity method. Among the three diversity methods used in this study, the results obtained with the spatial diversity method are presented and described using the 60-km data. Figure 7a shows one of the signals received across the 60-km range. During the experiment, a number of click sounds, presumed to be emitted by dolphins, were captured; these are indicated by red arrows in Figure 7a. More than 30,000 dolphins inhabit the East Sea [28], and the whistle sound made by these dolphins was recorded by the VLA; this suggested the presence of dolphins in the vicinity of the VLA during the experiment. In Figure 7a, the yellow box represents the BPSK signal. Because the click sound is an impulsive signal covering all frequencies, communication performance can be affected if it is included in the communication signal, as shown in Figure 7a. However, the effect of a small number of click sounds can be neglected. In this study, the effect of the click sounds was not considered, and their removal is outside the scope of the work presented herein. Therefore, three data packets that failed to decode owing to contamination by a large number of click sounds were excluded from the analysis.

(6) $$Q-\mathrm{function}\;:\;\;q\left(t\right)=\sum_{i=1}^{M,\;L,\;\mathrm{or}\;N}h_{i}\left(t\right)*h_{i}\left(-t\right).$$

The Q-function, as defined in Equation (6), is a metric used to assess the performance of the diversity method [6] and is expressed as the sum of the autocorrelation between channel impulse responses. As the order of diversity increases, the result of the Q-function more closely approximates that of the delta function, and the performance of the diversity method improves. The result of the Q-function is shown in Figure 7b for different orders of spatial diversity, where the result of each Q-function is the average over 15 packets. In Figure 7b, the blue, red, and black lines are averaged Q-functions when the order of diversity is 1, 8, and 16, respectively. The mainlobe of the Q-function is similar for the three orders of spatial diversity, but as the order of diversity increases, the sidelobes decrease and converge to zero.

**Figure 8:**
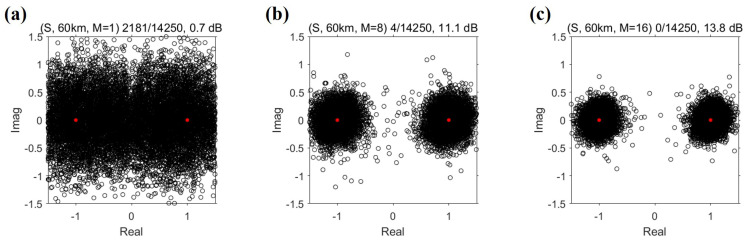
Scatter plots according to the order of spatial diversity (*M*): (**a**) M=1, (**b**) M=8, and (**c**) M=16. As the order of spatial diversity increases, the communication performance, in terms of BER and output SNR, improves. This is consistent with the sidelobe variation of the averaged Q-function.

The convergence of the Q-function to the delta function means that the TRP output using the diversity method is close to the source signal from Equation (1). In other words, the BER and output SNR can be improved, as shown in Figure 8. The output SNR, $SNR_{o}$, is defined as the reciprocal of the mean-square error between the information symbols and estimated symbols, as in Equation (7) [6].

(7) $$SNR_{o}=1/E{\left|{\vec{e}}_{k}\right|}^{2}=1/E{\left|I_{k}-{\hat{I}}_{k}\right|}^{2},$$

where $I_{k}$ and ${\hat{I}}_{k}$ are the $k_{th}$ information symbols and estimated symbols, respectively, and *E* denotes expectation. ${\vec{e}}_{k}$ is the difference between $I_{k}$ and ${\hat{I}}_{k}$, which is the noise for the $k_{th}$ symbol. As the source signal used in this study was modulated with binary-phase shift keying (BPSK), the number of bits and symbols are the same.

The noise power is equal to the denominator in Equation (7). For BPSK, as the information symbols are on the unit circle, the source power is one and is the same as the numerator in Equation (7). That is, the output SNR is related to the distance between the information symbols and estimated symbols in the scatter plot. If the estimated symbols are close to the information symbols, the output SNR is high. Figure 8a–c show scatter plots for three values of the order of spatial diversity: 1, 8, and 16, respectively. Figure 7 and Figure 8 indicate that, as the order of diversity increases, the BER and the sidelobe of the Q-function decreases and the output SNR increases. These results are consistent with the results reported in [6]. When the maximum order of diversity in the spatial diversity method was used, error-free performance was achieved.

### 4.3. Performance Comparison Using Various Diversity Methods

**Figure 9:**
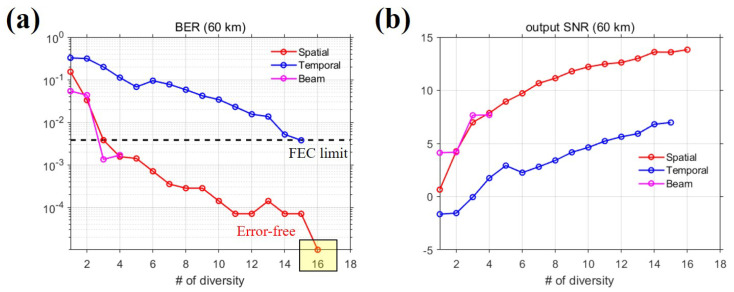
Variation in performance as a function of the order of diversity: (**a**,**b**) present the BER and output SNR results for the three diversity methods, respectively. The red, blue, and magenta solid lines with circles as markers represent the spatial, temporal, and beam diversity methods, respectively. To evaluate communication performance, the FEC limit ($3.8\times 10^{-3}$) is indicated by the black dashed line with the BER result. The spatial diversity method produced the best performance.

This section presents and compares the communication performance results according to the diversity method used. Because the diversity method depends on the channel variability, the performance may vary even for the same order of diversity. This implies that the performance would vary depending on which diversity method is selected. Figure 9 shows the variation in communication performance as the order of diversity increases for the three diversity methods. Figure 9a,b are the BER and output SNR results, respectively. Similar to the results presented in Section 4.2, in general, the BER decreased and the output SNR increased as the order of diversity increased for all methods. However, the performance depended on the diversity method. First, when the order of diversity is unity, the beam diversity method outperforms the spatial and temporal diversity methods. As mentioned in Section 2, the beam diversity method combines beamformed signals rather than received signals. Beamforming the received signal to the angle of the path can mitigate the effect of multipath time delay, which is known as spatial filtering [25,26,29]. When the order of diversity is unity, the effect of multipath time delay is not as strong as for the other diversity methods, and thus, the beam diversity method exhibits the best performance. However, because the beam diversity method does not have a large order of diversity, the difference in performance between an order of diversity of unity and the maximum order of diversity, is smaller than that observed for the other methods. In addition, the temporal and beam diversity methods exhibited similar performance in terms of output SNR (Figure 9b) based on when the maximum order of diversity was used, and the difference in output SNR was less than approximately 1 dB. However, in terms of BER (Figure 9a), the BER of the temporal diversity method was twice that of the beam diversity method [temporal diversity: 57/15,200 (=$3.75\times 10^{-3}$), beam diversity: 24/14,250 (=$1.68\times 10^{-3}$)]. Because the other organizations participating in the experiment designed communication signals with channel coding, we designed a communication signal that did not employ channel coding. Therefore, although the result of channel coding cannot be displayed, the black dashed line in Figure 9a shows the limit ($3.8\times 10^{-3}$) of the forward error coding (FEC) scheme, which is a standard practice in undersea systems for evaluating the performance of communication [30]. For the spatial and beam diversity methods, a BER lower than the FEC limit [31] was achieved when the order of the diversity was three or more. At the maximum order, the BER for the temporal diversity method was somewhat lower than the FEC limit. If the FEC scheme can be used when the order of diversity is at its maximum, all diversity methods will provide error-free performance.

**Figure 10:**
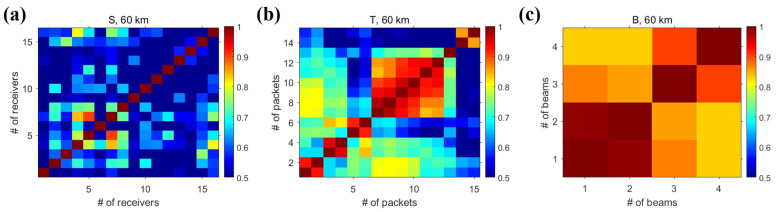
Co-diversity interference, $Q_{ij}$, for the three diversity methods. All plots show the correlation coefficient between the channel impulse responses estimated from two sets of data, i.e., the data obtained by two receivers from the point of view of the spatial diversity. The off-diagonal elements of $Q_{ij}$ represent the channel variability within the diversity scheme. (**a**–**c**) show the $Q_{ij}$ of the spatial, temporal, and beam diversity methods, respectively.

These results can be interpreted using the co-diversity interference matrix shown in Figure 10. In this study, we defined and utilized the co-diversity interference matrix as a metric representing the channel variability between the two diversities (e.g., two receivers in the spatial diversity method). Each element of this matrix is a correlation coefficient of two channel impulse responses within each diversity method:

(8) $$\mathrm{Co}-\mathrm{diversity}\;\;\mathrm{interference}\;\;\mathrm{matrix}\;:\;\;Q_{\mathrm{ij}}=\max\left(h_{i}\left(t\right)*h_{i}\left(-t\right)\right)\;\;\left(1\leq i,\;j\leq M,\;L,\;\mathrm{or}\;N\right).$$

Because the diagonal element of $Q_{ij}$ is itself, it has a maximum value (=1), and the off-diagonal elements represent channel similarity between different receivers (or times, beams) in the diversity method. The closer the value of the co-diversity interference matrix is to unity, the smaller the change in the channel.

The off-diagonal term in Figure 10 is related to channel variability; the smaller is the value in the off-diagonal, the greater is the channel variability. In the spatial diversity method, the number of off-diagonal term elements lower than 0.5 is greater than that of the other two diversity methods; therefore, in the BLAC18 environment, the channel variability in terms of space is the greatest. Combining the TRP outputs with high variability increases the extent to which the sidelobes decrease. Therefore, the spatial diversity method was superior to the other methods. Figure 9 shows that the performance of the beam diversity method is best when the order of diversity is unity owing to the effect of the spatial filter. However, from the viewpoint of channel variability, the performance improvement with the beam diversity method is the least because of the small channel variability. According to the channel variability results, the channel variability of the temporal diversity method is between that of the spatial diversity and beam diversity methods, and as a consequence, the range of performance change is the second largest after that of the spatial diversity method. However, despite having the second largest variation, when the order of diversity is at its maximum, the performance of the temporal diversity method is inferior to that of the beam diversity method, indicating that the spatial factor plays a greater role than time. In reality, when operating underwater objects with a short aperture, the temporal diversity method may be more effective than spatial or beam diversity methods, although the data rate will be reduced. In other words, a trade-off exists between the data rate and the order of diversity.

**Figure 11:**
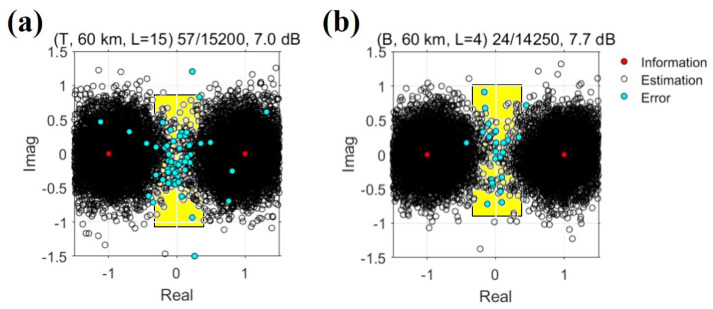
Performance comparison of the temporal and beam diversity methods when the order of diversity is maximum; (**a**,**b**) show the performance of the beam and temporal diversity methods, respectively. The yellow boxes indicate the area around the origin, and cyan filled circles indicate symbols corresponding to errors. In this experiment, the beam diversity method, which utilizes the features of the spatial filter, outperformed the temporal diversity method.

Figure 11 shows scatter plots of the temporal and beam diversity methods when the maximum order of diversity is utilized. In Figure 11, the red-, white-, and cyan filled circles represent information symbols, estimation symbols, and error symbols, respectively, and the yellow boxes represent the area around the origin. In Figure 11a,b, more symbols are close to the origin, which is the effect of the lowered SNR. The BPSK modulation technique is determined according to the sign of the real value of the symbol; hence, if many symbols have a real value close to the origin, the probability of an error increases. Because the output SNR of the temporal diversity method is lower than that of the beam diversity method, the estimated symbols were distributed more widely from the information symbols, and the number of error bits increased relatively. Most of the error symbols (i.e., cyan-filled circles) were confirmed to appear around the origin.

## 5. Conclusions

In this paper, we reported the first long-range underwater acoustic communication experiment (60 km range) in the East Sea, which was conducted in 2018, and analyzed the communication performance results for various TRP-based diversity methods (spatial, temporal, and beam). As the performance of the diversity method depends on channel variability, even if the order of diversity is the same, a performance difference occurs according to the type of method. For the minimum order of diversity (=1), the performance of the beam diversity method using beamformed signals, which mitigate the effect of multipath time delay, was the best in terms of the two metrics (BER and output SNR). For the maximum order of diversity, the performance of the spatial diversity method was the best among the three diversity methods in that the output SNR with this method was the highest. Furthermore, from the viewpoint of the BER, the performance was error-free only with the spatial diversity method. This is because the order of diversity is large, and the channel variability in terms of space is the greatest. For BLAC18 data, due to the small channel variability, the performance of the temporal diversity method was lower than that of the spatial diversity method. If the interval between data packets increases, the performance of the temporal diversity method can be further improved, but the data rate reduces, indicating a trade-off between the order the diversity and data rate. In practice, nevertheless, for underwater objects (e.g., submarines) with a short aperture, the temporal diversity method would be more efficient than the spatial and beam diversity methods. The results of the temporal and beam diversity methods are not error-free for the maximum order of diversity, but the BERs of these two methods are less than 1%, demonstrating the feasibility of long-range underwater acoustic communication in the East Sea.

## Data Availability

Not applicable.
